# Supplementary material for: Complexome profiling of the Chlamydomonas psb28 mutant reveals TEF5 as an early PSII assembly factor
Source: Plant Cell. 2025 Mar 17;37(6):koaf055. doi: 10.1093/plcell/koaf055 (PMC12164590; doi:10.1093/plcell/koaf055)
Supplement: koaf055_Supplementary_Data [file koaf055_supplementary_data.zip › psb28_interactive.zip!/plots/intensity/qualityPlots/peptides/ENR1_Cre06.g294950.t1.1.html]

### Cre06.g294950.t1.1

Trivial name: ENR1  
SeqCoverage [median]: 38.50  
Mass: 39.25 kDa  
  
 

| Sequence | MissedCleavage | Mass [Da] | wtIntensity | muIntensity | Score |
| --- | --- | --- | --- | --- | --- |
| AALESDTR | 0 | 861.42 | 7744.46 | 9310.95 | 178.87 |
| AIGFIDDMIR | 0 | 1149.59 | 1270.94 | 19679.39 | 92.44 |
| AWTVSEVAESVKK | 1 | 1432.76 | 2824.80 | 7509.33 | 78.76 |
| DSGNIDILVHSLANGPEVVKPLLEVSR | 1 | 2870.55 | 659.92 | 0.00 | 31.17 |
| FGPLMNEGGAVISLTYNASNR | 0 | 2210.08 | 2020.20 | 0.00 | 74.33 |
| KLSDGSLMTFSHIYPMDAVFDTMADVPEEVATNKR | 2 | 3914.85 | 0.00 | 3094.20 | 48.83 |
| VLAYEAGR | 0 | 877.47 | 2650.98 | 2720.37 | 95.35 |
| VNTISAGPLGSR | 0 | 1170.64 | 7385.56 | 12179.43 | 145.09 |
| YSYENSPIQK | 0 | 1227.58 | 4137.73 | 2632.31 | 182.39 |
|  |  |  | 28694.6 | 57126.0 |  |
